# Depicting individual responses to physical therapist led chronic pain self-management support with pain science education and exercise in primary health care: multiple case studies

**DOI:** 10.1186/s40945-017-0032-x

**Published:** 2017-04-20

**Authors:** Jordan Miller, Joy C. MacDermid, Julie Richardson, David M. Walton, Anita Gross

**Affiliations:** 1 School of Rehabilitation Therapy, Queen’s University, Kingston, 31 George Street, Kingston, Ontario K7L 3N6 Canada; 2 School of Physical Therapy, Western University, Room 1440, Elborn College, London, Ontario N6G 1H1 Canada

**Keywords:** Chronic pain, Self-management, Exercise, Pain education, Multiple case reports

## Abstract

**Background:**

Previous evidence suggests self-management programs for people with chronic pain improve knowledge and self-efficacy, but result in small to negligible changes in function. The purpose of this multiple case studies design was to describe the unique responses of six participants to a new self-management program aimed at improving function, to detail each component of the program, and to explore potential explanations for the varied trajectories of each of the participants.

**Case Presentation:**

Six participants who had been experiencing chronic pain for at least 5 years were included. All participants were enrolled 6 weeks of ChrOnic pain self-ManageMent support with pain science EducatioN and exercise (COMMENCE). Participants completed an assessment at baseline, 7 weeks (1-week follow-up), and 18 weeks (12-week follow-up). Each participant had a unique initial presentation and goals. Assessments included: function as measured by the Short Musculoskeletal Function Assessment – Dysfunction Index, how much participants are bothered by functional difficulties, pain intensity, fatigue, pain interference, cognitive and psychological factors associated with pain and disability, pain neurophysiology, self-efficacy, satisfaction, and perceived change. The self-management program was 6-weeks in length, consisting of one individual visit and one group visit per week. The program incorporated three novel elements not commonly included in self-management programs: pain neurophysiology education, individualized exercises determined by the participants’ goals, and additional cognitive behavioural approaches. Participants were all satisfied with self-management support received. Change in function was variable ranging from 59% improvement to 17% decline. Two potential explanations for variances in response, attendance and social context, are discussed. Several challenges were identified by participants as barriers to attendance.

**Conclusions:**

A primary care self-management intervention including pain education and individualized exercise has potential to improve function for some people with chronic pain, although strategies to improve adherence and reduce barriers to participation may be needed to optimize the impact.

**Electronic supplementary material:**

The online version of this article (doi:10.1186/s40945-017-0032-x) contains supplementary material, which is available to authorized users.

## Background

Primary health care often includes a diverse team of health-care providers and services working towards the ultimate goal of “better health for all” [1]. The role of physiotherapists in primary health care is gaining attention in recent years [2–6]; however, physical therapy in primary health care has a history dating back to the 1970s when physiotherapists adopted primary care roles in the United States Army [7]. Since that time, numerous studies have provided evidence that physiotherapists can provide quality, cost-effective primary care [3, 8–11].

Chronic conditions are among the most common reasons for a visit to a primary health care provider and chronic pain and musculoskeletal conditions, specifically, are among the most significant contributors to years lived with disability [12]. The prevalence of these conditions is expected to rise with an aging population and physiotherapists working in these settings need to be prepared to work with people experiencing multiple morbidities and complex health challenges.

Self-management support has received global attention as a potential response to the rise in chronic conditions in primary health care [13]. Evidence on the effectiveness of self-management programs for people experiencing chronic pain is limited. Most research investigating the effects of self-management on pain and disability has included people with either arthritis or low back pain. There is a dearth of literature including more diverse populations of people with chronic pain [14]. Systematic reviews on the effectiveness of self-management for low back pain and osteoarthritis suggest improvements in knowledge and self-efficacy, but small or negligible effects on function in comparison to usual care, wait-list, or attention-controls [14–16].

Physiotherapist involvement in the development and delivery of self-management programming appears to be growing [17]. Given the important role physiotherapists can play in improving function [18], involving physiotherapists in self-management programs provides an opportunity to target function and improve functional outcomes. Three treatment approaches within the scope of physical therapy practice that contribute to improvements in function are: pain neurophysiology education [19–21], applying cognitive behavioural principles [22, 23], and individualized, goal-oriented exercises [24–27]. Despite some evidence of improved function with these approaches, they have not been consistently incorporated into self-management supports.

The aim of this multiple case studies design was to describe the unique responses of six participants to a new self-management program aimed at improving function, to detail each component of the program, and to explore potential explanations for the varied responses of each of the participants. The cases were selected to demonstrate a range of effects sizes (low versus high effect exemplars) and to highlight barriers and facilitators to participation. The intervention described and evaluated in this study was ChrOnic pain self-ManageMent support with pain science EducatioN and exerCisE (COMMENCE). The innovative aspects of COMMENCE included incorporating pain neurophysiology education, cognitive-behavioural principles, and individualized, goal-oriented exercise within a self-management program that was delivered in a primary health care setting targeting a marginalized population of people with barriers to accessing healthcare.

The aim of this multiple case studies design was to describe the unique responses of six participants to a new self-management program aimed at improving function, to detail each component of the program, and to explore potential explanations for the varied responses of each of the participants.

## Case Presentation

Case studies aim to “investigate a contemporary phenomena within its real-life context” [28]. They allow for an in depth description of new health care interventions while considering the context in which the interventions are delivered and the impact these contexts can have on an individual’s outcome. Also, describing multiple case studies together allows for exploration of differences and similarities between cases [29]. The purpose and nature of the comparisons between cases is a fundamental element of multiple case studies and should be set when selecting cases and constructing the study design.

This case series included six participants recruited at Woodstock and Area Community Health Centre (WACHC) in Ontario, Canada. All participants were referred by a health care provider at WACHC. WACHC provides care to priority populations with barriers to accessing healthcare, including people with: addictions concerns, mental health challenges, low income, lack of health insurance, and isolated seniors. Importantly, this sample represents a population of people with chronic pain and multiple morbidities often excluded from treatment and research by barriers to accessing health care.

Included participants were adults who had been experiencing non-cancer related pain for at least 5 years. They did not meet any of the exclusion criteria: cancer related pain, medical “red flags” suggestive of a non-neuromusculoskeletal etiology of symptoms, casted fracture within the last 12 weeks, surgery within the last 26 weeks, and evidence of upper motor neuron lesion.

The six participants were selected from the first 18 participants who participated in the COMMENCE interventions. They were selected to represent the variance seen in this group of 18 participants based on their varied responses (clinically meaningful change versus no change exemplars) as well as their varied attendance (high versus low attendance exemplars) to the intervention. Table 1 presents the demographic characteristics of each participant at baseline. The participants selected can be visualized as three pairs: Participants 1 and 2 completed 9/12 visits and experienced no clinically meaningful changes at 12-week follow-up. Participants 3 and 4 experienced barriers to participating in the self-management program and discontinued participation after two or fewer visits. Participants 5 and 6 completed at least 10/12 visits and experienced several clinically meaningful improvements at 12-week follow-up.

**Table 1:** Baseline demographic information

Pair	High attendance, little change		Low attendance		High attendance, positive change	
Participant	1	2	3	4	5	6
Age	48	36	47	51	49	45
Sex	Male	Female	Female	Male	Male	Female
Education	High school diploma	High school diploma	High school diploma	High school diploma	Less than high school diploma	Less than high school diploma
Duration of pain	5 years	12 years	20 years	5 years	31 years	28 years
Area of pain	Primary concern: left hip	Primary concern: widespread pain including - headaches, bilateral shoulders, wrists, lower back and legs	Primary concern: low back with referral into legs	Primary concern: left shoulder, arm, wrist, and hand	Primary concern: low back with referral into legs	Primary concern: bilateral shoulders
	Secondary concerns: neck, right knee		Secondary concern: upper back and neck	Secondary concern: headaches, right shoulder, arm, and hand	Secondary concerns: headaches, neck, bilateral shoulder, arm, hand, foot, and ankle	Secondary concerns, left elbow, wrist, and hand, lower back, bilateral hips and knees
Diagnosis reported by participant	OA	FM	Disc herniation	FM, CRPS	No diagnosis	FM, OA
Medications	Acetaminophen, Gabapentin, Oxycodone, Percocet,	Celexa, Gabapentin, Lorazapam, Methadone, Olanzapine	Bisoprolol, Carbamazepine, Celecoxib, Clonazepam, Domperidone, Gabapentin, Mirtazapine, Tolterodine, Venlafaxine	Bisoprolol, Crestor, Cymbalta, Diclofenac, Hydromorphone, Plavix, Rabeprazole,	None	Celebrex, Cymbalta, Duvoid, Hydrochlorothiazide, Propranolol, Quetiapine
Comorbidities	None	Anxiety, Depression, PTSD	Anxiety, Depression, Hypertension, Urinary incontinence	Depression, Hypertension, Gastric reflux	None	Depression, Diabetes, Hypertension

*OA* Osteoarthritis, *FM* Fibromyalgia, *CRPS* Complex regional pain syndrome, *PTSD* Post-traumatic stress disorder

### Examination

Participants completed assessments at baseline, 7 weeks (1 week after completion of the 6-week intervention), and 18 weeks (12 weeks after the end of the intervention). Demographic and clinical information was collected at baseline. Self-reported outcome measures were completed at all time-points. Additionally, participants participated in a thorough history and physical examination with a physiotherapist including screening for red-flags, neurological assessment, strength testing, range of motion assessment, and functional movement assessment.

#### Demographic and clinical information

The following information was collected at baseline: age, sex, length of time since symptom onset, diagnosis provided by a medical professional as reported by the patient, medication use, and comorbidities.

#### Self-report measures

The primary outcome was function as measured by the Short-Musculoskeletal Function Assessment – Dysfunction Index (SMFA-DI) [30]. Secondary outcomes included: Short Musculoskeletal Function Assessment – Bother Index (SMFA-BI) [30], Numeric Pain Rating Scale (NPRS) [31], Numeric Fatigue Rating Scale (NFRS) [32], PROMIS Pain Interference Item Bank - 8 items [33], Pain Catastrophizing Scale (PCS) [34–36], Tampa Scale of Kinesiophobia - 11 (TSK-11) [37], Injustice Experience Questionnaire (IEQ) [38], Neurophysiology of Pain Questionnaire (NPQ) [39], Pain Self Efficacy Questionnaire (PSEQ) [40–42], Patient Health Questionnaire – 9 (PHQ-9) [43–45], Post-traumatic Stress Disorder Checklist – civilian version (PTSD-C) [46, 47], global perceived effect, patient satisfaction, and patient expectations for recovery. Expectations for recovery were assessed with two questions: i) Do you think your pain will improve? ii) Do you think your functional abilities will improve? Table 2 shows the construct measured by each outcome measure, the range of each scale, and the minimal important change for each measure. Table 3 describes the baseline scores on each measure for each participant.

**Table 2:** Outcome measures and potential predictors of treatment response

Construct	Outcome Measure	Scale range	Minimal important difference
Function	Short Musculoskeletal Function Assessment – Dysfunction Index (SMFA-DI)	0-100	7.5 points^a^
How much participants are bothered by difficulty with functional activities	Short Musculoskeletal Function Assessment – Bother Index (SMFA-BI)	0-100	10.5 points^a^
Pain Intensity	Numeric Pain Rating Scale (NPRS)	0-10	2 points [65]
Fatigue	Numeric Fatigue Rating Scale (NFRS)	0-10	1.4 points [66]
Pain Interference	PROMIS Pain Interference Item Bank - 8 items	8-40	5 points^a^
Catastrophic thinking	Pain Catastrophizing Scale (PCS)	0-52	38% of scale [35]
Fear of symptom exacerbation	11-item Tampa Scale of Kinesiophobia (TSK-11)	11-44	5.6 points [67]
Sense of perceived injustice	Injustice Experience Questionnaire (IEQ)	0-48	7 points^a^
Pain neurophysiology knowledge	Neurophysiology of pain test (NPT)	0-13	1.1 points^a^
Self efficacy	Pain Self Efficacy Questionnaire (PSEQ)	0-60	11 points [68]
Depressive symptoms	Patient Health Questionnaire - 9 (PHQ-9)	0-27	5 points [44]
Post-traumatic stress symptoms	Post-traumatic Stress Disorder Checklist –Civilian Version (PCL)	17-85	8.5 points^a^

^a^In the absence of an established MCID or MDC, this case series considered half a standard deviation as a minimally important difference as has been identified as a common MCID for quality of life measures [69]. In these instances, clinical data from Woodstock and Area Community Health Centre was used to establish the standard deviation

**Table 3:** Baseline Scores for participants

Participant	1	2	3	4	5	6
SMFA-DI (0–100)	45.6	56.6	70.6	51.5	61.0	51.5
SMFA-BI (0–100)	45.8	75.0	87.5	41.7	68.2	31.3
NPRS (0–10)	8	7	9	7	9	8
NFRS (0–10)	7	9	9	8	8	9
PHQ-9 (0–27)	20	21	26	13	20	22
PI (8–40)	40	36	36	32	24	31
PCS (0–52)	43	45	48	31	34	28
TSK-11 (11–44)	36	31	29	30	22	31
IEQ (0–48)	31	45	28	27	29	34
PCL-C (17–85)	58	78	70	38	45	62
PSEQ (0–60)	15	6	25	29	40	35
Pain expectations (yes/no/unsure)	Unsure	Unsure	Unsure	Yes	Unsure	Yes
Function expectations (yes/no/unsure)	Unsure	Unsure	Yes	Yes	Unsure	Yes

abbreviations: SMFA = short musculoskeletal function assessment, DI = dysfunction index, BI = bother index, NPRS = numeric pain rating scale (average pain intensity over the past 2 weeks), NFRS = numeric fatigue rating scale (average fatigue over the past 2 weeks), PHQ-9 = 9-item Patient health questionnaire, PI = 8-item PROMIS pain interference scale, PCS = pain catastrophizing scale, TSK-11 = 11-item Tampa Scale of Kinesiophobia, IEQ = Injustice Experience Questionnaire, PCL-C = Post-traumatic stress disorder checklist – Civilian Version, PSEQ = Pain self-efficacy questionnaire, pain expectations = Do you think your pain will improve? y = yes; n = no; u = unsure, Function expectations = do you think your functional abilities will improve? y = yes; n = no; u = unsure

#### Adverse events or harms

The physiotherapist asked participants at each individual visit about adverse events associated with the intervention.

### Intervention

ChrOnic pain self-ManageMent with pain science EducatioN and exerCisE (COMMENCE) was a 6 week program that included two sessions per week with a physiotherapist. The first session each week was in a group setting. This session included education on pain science, cognitive-behavioural approaches, and self-management strategies. The second session was an individualized, one-to-one session including support for implementing self-management strategies and development of an individualized, goal-oriented exercise program. The registered physiotherapist implementing COMMENCE had a Master’s of Science in physiotherapy, 4 years of clinical experience working with people living with chronic pain, and post-graduate training in cognitive and behavioural approaches to chronic pain management. Additional file 1 describes the weekly objectives and Additional file 2 provides a rationale for each of the included treatment strategies.

#### Group pain science and self-management education

Group sessions were interactive 1.5 h sessions with 3–5 people once per week for 6 weeks. The participants received pain science education including discussion about the function of the nervous system, changes in multiple body systems when pain persists, neuroplasticity, the relationship between physical activity and pain, and the influences of stress, thoughts and emotions on pain. Self-management strategies focused on applying the information learned with the goal of increasing activity levels and participation in life role activities while controlling symptoms. Participants were given a workbook that they brought to each appointment to track participation and allow problem solving to overcome any potential barriers to implementation.

#### Individualized self-management and exercise

The 30–45 min individualized sessions varied between individuals and were delivered once per week for 6 weeks. The individual sessions allowed for discussion about personal implementation plans for self-management strategies learned in the group session. Also, the physiotherapist worked with the patient to develop an individualized exercise program aimed at working towards patient-specific goals. The tailoring of exercises to individual patients involved a series of questions that the physiotherapist asked the participant. First, the physiotherapist asked the participant to explore movements of the painful area of the body to determine which movements the participant could perform without increasing pain. The participant was encouraged to perform 6–8 repetitions of these movements, frequently throughout the day. Second, participants were asked to consider potential barriers and facilitators to the short- and long-term function and participation goals they set at the beginning of the program. The therapist and participant then work together to develop an exercise program to help the participant enhance facilitators and minimize physical and cognitive barriers to participation through graded physical activity and exercise. Finally, the dosage was individualized by asking the participant to determine an amount of the exercise or activity that does not result in an increase in pain 30–60 min after finishing the exercise.

#### Co-intervention

Participants were able to continue with co-interventions during the 18-week study period and co-interventions were monitored through a self-report diary at each data collection time-point. Three participants booked appointments with their family doctor during the study period (participants 1, 2, and 3). Participant 1 changed dosage of gabapentin, participant 2 had no changes in medications or other treatments, and participant 3 changed anti-depressant medications. There were no other co-interventions reported by participants.

### Outcomes

In order to help visualize the varied outcomes, the six participants will be referenced as three pairs. The first pair (participant 1 and 2) will be referred to as the “high attendance, no clinically meaningful change” pair. They both attended 9/12 scheduled sessions (75%). The second pair (participant 3 and 4) both experienced barriers to participating in the program and will be referred to as the “low attendance” pair. Participant 3 attended 2/12 sessions (17%) and participant 4 attended 1/12 sessions (8%). The third pair (participant 5 and 6) will be referred to as the “high attendance, clinically meaningful improvement” group. Participant 5 attended 11/12 sessions (92%) and participant 6 attended 10/12 sessions (83%). Attendance rates for group visits (20/36 visits) and individual visits (22/36 visits) were similar. Attendance and adherence to each self-management strategy are described in Table 4.

**Table 4:** Adherence to included self-management strategies at individual visits

Visit		Participant 1	Participant 2	Participant 3	Participant 4	Participant 5	Participant 6
1	Goal setting	No	Did not attend	Yes	Yes	Did not attend	Yes
	Frequent pain-free movement (completion = ≥ 3 times/day)	Yes		Yes	Yes		Yes
	Goal-specific exercises (completion = ≥ once/day)	Yes		Yes	No		Yes
	Activity log	No		Yes	No		Yes
2	Goal setting	Yes	Yes	Did not attend	Did not attend	Yes	Did not attend
	Frequent pain-free movement (completion = ≥ 3 times/day)	Yes	Yes			Yes	
	Goal-specific exercises (completion = ≥ once/day)	Yes	No			Yes	
	Activity schedule and log	Yes	No			Yes	
	Graded activity plan	Yes	No			Yes	
3	Frequent pain-free movement (completion = ≥ 3 times/day)	Yes	Yes	Did not attend	Did not attend	Yes	Yes
	Goal-specific exercises (completion = ≥ once/day)	No	Yes			Yes	Yes
	Activity schedule and log	No	Yes			Yes	Yes
	Graded activity plan	Yes	Yes			Yes	Yes
	Breathing (completion = ≥ once/day)	Yes	Yes			Yes	Yes
	Relaxation (completion = ≥ once/day)	Yes	Yes			No	Yes
	Developed plan for improved sleep	No	Yes			No	Yes
4	Frequent pain-free movement (completion = ≥ 3 times/day)	Yes	Yes	Did not attend	Did not attend	Yes	Yes
	Goal-specific exercises (completion = ≥ once/day)	Yes	Yes			Yes	Yes
	Activity schedule and log	No	Yes			Yes	Yes
	Graded activity plan	Yes	Yes			Yes	Yes
	Breathing (completion = ≥ once/day)	Yes	Yes			No	Yes
	Relaxation (completion = ≥ once/day)	No	Yes			No	Yes
	Developed plan for improved sleep	Yes	Yes			Yes	Yes
	Positive self-talk	Yes	No			No	Yes
	Thought monitoring log	No	No			No	Yes
5	Frequent pain-free movement (completion = ≥ 3 times/day)	Yes	Did not attend	Did not attend	Did not attend	Yes	Yes
	Goal-specific exercises (completion = ≥ once/day)	No				Yes	Yes
	Activity schedule and log	No				Yes	No
	Graded activity plan	Yes				Yes	Yes
	Breathing (completion = ≥ once/day)	Yes				No	No
	Relaxation (completion = ≥ once/day)	No				No	No
	Positive self-talk	No				No	Yes
	Thought monitoring log	No				No	Yes
	Developed a flare up plan	Yes				Yes	Yes
6	Frequent pain-free movement (completion = ≥ 3 times/day)	Did not attend	Yes	Did not attend	Did not attend	Yes	Yes
	Goal-specific exercises (completion = ≥ once/day)		Yes			Yes	Yes
	Activity schedule and log		Yes			Yes	No
	Graded activity plan		Yes			Yes	Yes
	Breathing (completion = ≥ once/day)		Yes			No	Yes
	Relaxation (completion = ≥ once/day)		Yes			No	Yes
	Positive self-talk		No			No	Yes
	Thought monitoring log		No			No	Yes
	Developed a flare up plan		Yes			Yes	Yes

Completion of self-management strategy was recorded by the treating physiotherapist based on the combination of participant self-report and workbook completion

Missed sessions for participants 1, 2, 5, and 6 were due to illness (4), specialist medical appointments (2), forgotten appointments (1), and anxiety interfering with leaving the house (2). Participant 3 experienced an exacerbation of depression and was admitted to hospital for suicidal ideations after two sessions. Participant 4 attended one session before a change in job that resulted in extended hours of work and the decision not to continue participation.

**Fig. 1:**
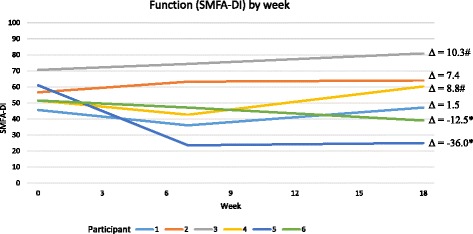
Change in function over time by participant. SMFA-DI = Short musculoskeletal function assessment – Dysfunction Index; Assessment time points = 0 weeks (before intervention), 7 weeks (1-week after intervention), and 18 weeks (12 weeks after intervention). * = clinically meaningful improvement in function, # = clinically meaningful decline in function

The participants demonstrated variable change in the primary outcome, function, throughout the program and follow-up period as measured by the SMFA-DI (see Fig. 1). At the 18-week assessment (12-week follow-up), neither of the two “high attendance, no clinically meaningful change” pair experienced any meaningful change in function; both of the “low attendance” pair experienced a clinically meaningful decline in function; and both of the “high attendance, positive change” pair experienced meaningful improvements in function.

The outcomes for each of the primary and secondary measures are presented in Table 5. Participant 1 experienced improvement in fatigue and fear of movement/re-injury, but increased pain at 12-week follow-up. Participant 2 had improved scores on fatigue, depressive symptoms, and self-efficacy at 12-week follow-up; however, she scored worse on measures of pain and function. Participant 3 reported a long-term improvement in fear of movement/re-injury and a long-term worsening of function. Participant 4’s scores on fear of movement/re-injury and sense of perceived injustice improved, while function had worsened from baseline. Participant 5 and 6 both demonstrated clinically meaningful changes in all outcomes at 12-week follow-up except fear of movement/re-injury for participant 5.

**Table 5:** Summary of outcomes

	High attendance, no meaningful change pair				Low attendance pair				High attendance, meaningful improvement pair			
Participant	1		2		3		4		5		6	
Assessment time-point (week)	7	18	7	18	7	18	7	18	7	18	7	18
SMFA-DI (0–100)	**-9.6** ^a^	−1.5	6.6	7.4	3.7	**10.3** ^b^	**−8.9** ^b^	8.8^a^	**−37.5** ^b^	**−36.0** ^b^	−4.4	**−12.5** ^b^
SMFA-BI (0–100)	**−12.5** ^b^	**−29.1** ^b^	**−20.8** ^b^	0.0	4.2	8.3^a^	**−16.7** ^b^	−2.1	**−49.4** ^b^	**−57.4** ^b^	**−10.5** ^b^	**−14.6** ^b^
NPRS (0–10)	1	2	1	3^a^	1	0	0	1	**−7** ^b^	**−5** ^b^	**−3** ^b^	**−5** ^b^
NFRS (0–10)	**−2** ^b^	**−2** ^b^	**−2** ^b^	**−2** ^b^	1	−1	−1	−1	**−5** ^b^	**−3** ^b^	**−2** ^b^	**−3** ^b^
PHQ-9 (0–27)	0	3	−3	**−10** ^b^	−2	−3	−3	0	**−18** ^b^	**−12** ^b^	**−9** ^b^	**−14** ^b^
PI (8–40)	**−8** ^b^	0	**−5** ^b^	−4	2	−1	**−13** ^b^	−2	**−12** ^b^	**−7** ^b^	**−12** ^b^	**−19** ^b^
PCS (0–52)	**−36** ^b^	−4	−6	−4	−10	−10	−10	−7	**−26** ^b^	**−17** ^b^	**−19** ^b^	**−24** ^b^
TSK-11 (11–44)	−2	**−6** ^b^	−1	−1	−3	**−7** ^b^	**−8** ^b^	**−6** ^b^	**−6** ^b^	−2	**−7**	**−7** ^b^
IEQ (0–48)	−6	−4	2	−1	13^b^	1	**−10** ^b^	**−8** ^b^	**−18** ^b^	**−15** ^b^	**−11** ^b^	**−23** ^b^
PCL-C (17–85)	**−24** ^b^	**−32** ^b^	**−14** ^b^	−2	−8	2	**−11** ^b^	−6	**−13** ^b^	**−19** ^b^	**−27** ^b^	**−32** ^b^
PSEQ (0–60)	**22** ^b^	9	5	11^b^	−7	7	4	9	6	5	−3	10
GPE (−3 to +3)	0	0	1	1	−1	0	1	1	2	2	1	1
Satisfaction (−3 to +3)	1		1		2		2		3		3	

^a^clinically meaningful improvement, ^b^clinically meaningful decline; abbreviations: SMFA = short musculoskeletal function assessment, DI = dysfunction index, BI = bother index, NPRS = numeric pain rating scale (average pain intensity over the past 2 weeks), NFRS = numeric fatigue rating scale (average fatigue over the past 2 weeks), PHQ-9 = 9-item Patient health questionnaire, PI = 8-item PROMIS pain interference scale, PCS = pain catastrophizing scale, TSK-11 = 11-item Tampa Scale of Kinesiophobia, IEQ = Injustice Experience Questionnaire, PCL-C = Post-traumatic stress disorder checklist – Civilian Version, PSEQ = Pain self-efficacy questionnaire, GPE = global perceived effect (−3 = much worse, −2 = moderately worse, −1 = slightly worse, 0 = no change, 1 = slightly better, 2 = much better, 3 = completely better, Satistfaction = patient reported satisfaction with health care (−3 = very dissatisfied, −2 = moderately dissatisfied, −1 = slightly dissatisfied, 0 = neutral, 1 = slightly satisfied, 2 = moderately satisfied, 3 = very satisfied)

Two participants (1 and 2) reported transient (<72 h) increases with pain after exercise or increases in activity with at least one session. Otherwise, there were no adverse events or side-effects reported.

## Discussion

People with chronic pain frequently suggest improved function is an important goal for treatment [48]. Also, reducing the financial burden of chronic pain requires improved ability to reduce disability [49–51]. Self-management support for chronic pain is an important opportunity to facilitate improvements in function and participation for people living with chronic pain [52]. This multiple case studies design study described the response of six individuals to chronic pain self-management support with pain science education and exercise (COMMENCE) that aimed to improve function for people with chronic pain and related disability. While the case series design does not allow comment on the effectiveness or efficacy of the intervention, it provides an opportunity to provide details on the COMMENCE intervention that is currently being evaluated in a randomized controlled trial (ClinicalTrials.gov, NCT02422459). It also provides an opportunity to discuss several observations from these case studies: the opportunity for physiotherapists to contribute to self-management programming through interventions targeting function, the large variance in response to self-management programs, and the multiple complex barriers to attendance that many people with chronic pain experience.

There has been discussion by self-management facilitators and researchers regarding peer-led versus professional led self-management programs [53, 54]. Qualitative evidence suggests that while participants in self-management programs view health care professionals as more knowledgeable, they do not necessarily view health care professional led programs as more valuable [54]. Also, it has been suggested that peer-led programs may help to build greater capacity for self-management support [53]. COMMENCE contains three treatment strategies that may not be delivered effectively by lay-persons: pain science education, cognitive behavioural principles, and individualized, goal-oriented exercises. A hypothesis which drove the development of COMMENCE was that physiotherapists are better positioned to implement these self-management approaches given their expertise in facilitating functional improvements in people with disabilities. A scoping review [17] identified seven previous studies that involved a physiotherapist in self-management support for chronic pain suggesting others may share this perceived value. Future research is likely to provide important evidence on differences between self-management support and functional interventions provided by health care providers versus lay-persons. A randomized controlled trial by Coleman and colleagues is currently underway comparing these two different delivery methods for self-management support [55].

While randomized trials describe the mean group effect, a multiple case study design provides the opportunity to visualize and analyze individual patient trajectories. In this case series, a large variance in individual responses is evident suggesting the potential to improve mean outcomes if the underlying reasons for suboptimal outcomes could be identified. The means of these six participants might suggest an overall 10% improvement in function at 1-week follow-up and a 5% improvement in function at 12-week follow-up. The clinical importance of these changes might be questioned. However, it is clear that two individuals experienced clinically important improvements in function, while others experienced no change or a decline in function. This is seen by the changes in function ranging from a 6.6 point from a 10.3 point (14.6%) decline in function to a 36.0 point (59.0%) improvement at 12-week follow-up. Similar variances in response were demonstrated with other outcomes (see Table 5). Importantly, the variance may represent differences in response to the treatment, differences in participation/adherence or fluctuations in self-reported function over time in this population of people with complex pain. Future research with a control group may provide valuable information regarding whether this variance is related to the intervention itself or the population being studied; and what additional factors may contribute to fluctuations independent of treatment.

One potential reason for the variance in response to the program is differences in treatment attendance or adherence. At 12-week follow-up, the two participants who attended less than 3/12 (25%) visits experienced a clinically meaningful decline in function, the two who attended 9/12 sessions experienced no change, and the two who attended 10 or more sessions experienced clinically meaningful improvement. A similar pattern can be visualized using the adherence to each strategy described in Table 4 and outcomes described in Table 5, where people who achieved greater adherence to the self-management strategies generally experienced improved outcomes. The variance in functional change could represent a dose–response relationship with COMMENCE. Alternatively, certain factors that make people more likely to attend and adhere could also make people more likely to experience improvements in function. These factors could be explored in secondary analyses within a randomized controlled trial. The multiple case studies design; however, allows for an exploration of detailed contextual factors that may contribute to individual patient trajectories that cannot be designed in clinical trials. The contexts of each individual patient could influence their individual outcome through interfering with or facilitating attendance or adherence, or through distinct mechanisms.

An example of the influence of social contexts can be seen by comparing the social contexts of the “high attendance, little change” pair (participant 1 and 2) with the “high attendance, positive change” pair (participant 5 and 6). Participant 1 and 2 both suggested their social contexts negatively influenced their self-management. Participant 1 reported challenges carrying out self-management skills and focusing on his own recovery because he was a committed caregiver for his partner. He suggested the stress of his caregiver responsibilities contributed to his pain and that it was difficult to focus on new self-management strategies given other responsibilities. His outcome measures suggest short-term improvement, but no change at 12-week follow-up. One possible explanation is that scheduled appointments allowed him to dedicate time to increases in physical activity and self-management, but it was difficult to prioritize these behaviors after the end of the program given his other responsibilities. It is worth noting the concordance of this finding with evidence suggesting a high prevalence of chronic pain in caregivers [56]. This highlights the need to measure adherence to self-management strategies and mediators of adherence both during the treatment period and during follow-up periods to fully understand uptake of these type of interventions.

Participant 2 was a woman with a long history of chronic pain, anxiety, depression, and post-traumatic stress disorder. She reported that group settings and certain social situations exacerbate her anxiety and post-traumatic stress and she cancelled two visits for this reason and rescheduled several others. Similarly, she reported having a very small social network due to her social anxieties. This context could relate to her chronic pain and pain-related disability as people with post-traumatic stress [57] and low levels of social support [58] are more likely to experience chronic pain and pain-related disability. While the group context is intended to increase social support, physiotherapists may need to identify patients with social anxiety and either provide individual treatment or preparatory treatment of the social anxiety to achieve a successful outcome.

In contrast, participants 5 and 6 reported that social supports contributed to their success with increasing functional abilities and participation in important life roles. Participant 5 lived with three brothers who were supportive of his increases in physical activity. Also, he reported taking on additional roles around his home throughout the program, which provided a sense of accomplishment. Similarly, participant 6 reported being surrounded by supportive family and friends, which contributed to her changes in function. She suggested her goals of being able to take her grandchildren to the park and coach one of her grandchildren in soccer positively influenced her participation and perseverance throughout the program. Also, she suggested that increasing her abilities allowed her to volunteer at her church, which was an important source of positive re-enforcement for the changes she was making. An interaction between social support and physiotherapeutic intervention facilitating greater role fulfilment is in keeping with a biopsychosocial view of rehabilitation.

The influences of social contexts and attendance on response to self-management programs are not mutually exclusive. This can be seen with the “low attendance” pair (participants 3 and 4). Participant 3 had high levels of pain and depression. While her scores on depression were very high at the start of the study, she did not report any suicidal ideations. However, after just 2 visits she separated with her husband. At this point, her depression worsened and she had suicidal ideations with a plan to carry out those ideations. At this time, she was referred to emergency psychiatric care at a local hospital. Her worsening pain and disability at this time was very likely influenced by her depression [59, 60]. Also, her depression interfered with participation in treatment, which reduced the chance that the treatment could influence outcomes. Participant 4 worked modified hours and duties in a produce department in a grocery store at the initial assessment. After just 1 visit, he took a new job as a produce manager at a different store. This transition involved an increase in work hours, so he did not feel comfortable devoting time to a 6-week treatment program during this period of change. His reduction in function throughout the treatment period could be due to the inability to participate, increased pain associated with the increased physical demands of work, or due to increased stress secondary to the responsibilities of his new job.

A key theme from this case series is that people with complex chronic pain experience challenges in attending health care visits. Missed appointments or discontinued treatment occurred due to mental health concerns, change in work status, illness, or conflicting health care appointments. Low attendance poses challenges for clinical practice as low adherence has been shown to predict poorer outcomes in self-management programming [61]. Some challenges with attendance were anticipated. Multiple morbidities are common in people with chronic pain and people with multiple morbidities frequently report difficulty with self-management and access to health care [62, 63]. However, the degree to which the social contexts influenced attendance and adherence was far greater than the team anticipated. This highlights an important learning opportunity and challenge for clinicians who work with people experiencing pain. Clinicians working with these populations need to be prepared to help participants problem solve to overcome barriers to attendance and to reschedule frequently to allow adequate treatment fidelity. Also, this may suggest working within a multidisciplinary team may be needed to help address the complex determinants of health in this population. For example, social workers may provide valuable contributions to addressing social factors and mental health care providers may help to address psychological factors that are associated with pain, disability, and difficulty participating in health care interventions.

Low attendance also makes it challenging for researchers to achieve acceptable retention rates in chronic pain research or to be confident that the treatment effects from trials demonstrating potential efficacy can be generalized to clinical practice in this population. Chronic pain is an important burden in a marginalized population of people with multiple morbidities, poverty, mental health concerns, or social isolation. Yet, this population is often under-represented in chronic pain research. This may be due to recruitment strategies involving health care facilities where there are often inequities in access [64]. Pain research in these marginalized populations is important to ensure generalizability of results; however, the low attendance in this case series helps identify a potential challenge in expanding pain research into these populations.

## Conclusion

The varied responses of participants and barriers to participation evident from the six cases described in this study are important considerations for physiotherapists working in primary health care settings. The self-management program detailed provides an example of how a physiotherapist can incorporate individualized exercise, pain science education, and cognitive behavioural principles into a self-management program with the goal of improving the function of participants.

## Additional files

Additional file 1: COMMENCE weekly objectives. (PDF 232 kb)

Additional file 2: Rationale for interventions, strategies, and objectives included. (PDF 304 kb)

## Abbreviations

BI: Bother Index
COMMENCE: Chronic Pain Self-Management Support with Pain Science Education and Exercise
DI: Dysfunction Index
IEQ: Injustice Experience Questionnaire
NFRS: Numeric Fatigue Rating Scele
NPQ: Revised Neurophysiology of Pain Questionnaire
NPRS: Numeric Pain Rating Scale
PCS: Pain Catastrophizing Scale
PHQ-9: 9-item Patient Health Questionnaire
PI: Pain Interference
PPT: Pressure Pain Threshold
PROMIS: Patient Reported Outcome Measurements Information System
PSEQ: Pain Self-Efficacy Questionnaire
PTSD-C: Post-Traumatic Stress Disorder Checklist – Civilian Version
SMFA: Short Musculoskeletal Function Assessment
TSK: Tampa Scale of Kinesiophobia
WACHC: Woodstock and Area Community Health Centre
