# Supplementary material for: Depicting individual responses to physical therapist led chronic pain self-management support with pain science education and exercise in primary health care: multiple case studies
Source: Arch Physiother. 2017 Apr 20;7:4. doi: 10.1186/s40945-017-0032-x (PMC5759926; doi:10.1186/s40945-017-0032-x)
Supplement: Supplementary file 1 — COMMENCE weekly objectives. (PDF 232 kb) [file 40945_2017_32_MOESM1_ESM.pdf]

Additional file 1: COMMENCE weekly objectives

Week	Self-management	Pain Education	Individualized exercise	Cognitive-behavioural principles
**1 -<br>Introduction to pain physiology, goal setting and exercise**	• Introduction to progressive goal setting by actively setting short and long-term goals<br>• Introduction to activity scheduling to plan and record activities each day	• Introduction to pain physiology<br>• Discuss biological, psychological, social influences on pain<br>• Breakdown the common misconception of a close relationship between tissue damage and pain through stories and metaphors demonstrating the complexity of pain<br>• Describe neuroplasticity and potential for changes in abilities with gradual increases in activity	• Frequent (every 2 hours) movement that doesn't increase pain as a tool for pain-relief and reduced sensitivity to movement<br>• Exercises that simulate functional tasks (1-2 times/day). Participants are encouraged to self-monitor intensity and volume with the instructions not to avoid pain at the time of exercise, but to avoid pain that lasts an hour afterwards	• Establish a strong therapeutic relationship in which the patient is on an 'equal level' with the health care provider<br>• Encourage disclosure<br>• Increase expectation of improvement in function through description of neuroplasticity and potential for change<br>• Begin to develop a sense of control over activities through active goal setting and activity planning
**2 -<br>Movement and neuroplasticity**	• Review activity log and use activity schedule to plan for upcoming week<br>• Collaborate on a graded activity plan to work towards one of goals set in previous week	• Briefly review pain physiology from week 1<br>• Discuss influence of pain on movement and movement on pain<br>• Describe how frequent movement can facilitate increases in activity participation<br>• Review neuroplasticity and how we can use movement strategies to encourage changes in pain and functional abilities	• Reflection on exercises from first week, problem solving barriers to successful performance, recognizing successes<br>• Use the reflection on week 1 to modify, maintain, or progress exercises covered during first week<br>• Add 1-3 goal oriented exercises determined collaboratively<br>• Develop plan for frequent aerobic activity	• Continue to build expectation of improvement in function by discussing the potential for movement to improve pain and function<br>• Develop abilities and confidence necessary to create a plan to work towards goals<br>• Empower the patient with exercises that can be performed without an increase in symptoms
**3 –<br>The complex relationships between stress and pain**	• Review activity log and progress towards goals<br>• problem solve barriers to goals set in the previous week<br>• Establish activity and participation goals for third week<br>• Introduce novel breathing and relaxation strategies<br>• Develop a plan to utilize relaxation and other stress reducing activities<br>• Discuss strategies for improved sleep	• Briefly review the influence of pain on movement and movement on pain<br>• Discuss the relationship between stress and pain using stories, metaphors, and evidence to clearly depict the relationship<br>• Describe the interaction of the nervous system, endocrine system, and immune systems in response to stress and pain<br>• Discuss positive influences on stress response systems (regular exercise, relaxation, enjoyable activities, social supports)	• Reflect on exercises from week 2<br>• Use the reflection on week 2 to modify, maintain, or progress exercises<br>• Encourage participant to consider which exercises are ready for progression<br>• Review plan for frequent aerobic activity and modify plan if needed<br>• Develop plan to implement physical activities that have been practiced with exercise into daily life	• Providing people with an explanation of the physiological link between stress and pain may help to validate their symptoms and help them feel understood<br>• Continue to develop a sense of control over symptoms and activity levels with increases in activity participation<br>• Increasing number of 'tools' to utilize when experiencing increases in symptoms may increase confidence in trying new activities or resuming discontinued activities
**4 –<br>Thoughts, emotions and pain**	• Review activity log and progress towards goals during third week<br>• Problem solve any barriers to	• Briefly review the relationships between activity, stress and pain<br>• Discuss how both positive and	• Reflect on exercises from week 3<br>• Use the reflection on week 3 to modify, maintain, or progress	• Validate participant's negative thoughts and relationship with pain

	goals from third week<br>• Establish activity and participation goals for fourth week<br>• Review stress reduction strategies and assess successes and barriers<br>• Provide thought monitoring tools<br>• Introduce positive self-talk<br>• Develop plan to implement new strategies	negative thoughts can influence stress and pain using physiology, stories and metaphors<br>• Discuss the relationship between emotions and pain<br>• Discuss strategies to improve mood: increases in activity, reflecting on positive change, engaging with support networks, and enjoyable activities	exercises<br>• Encourage participant to consider how many total exercises is realistic for part of a long-term daily routine and add exercises if needed/able<br>• Review plan for frequent aerobic activity and modify plan if needed	• Provide strategies to reduce impact of negative thoughts (thought monitoring and self-talk)<br>• Encourage reflection on changes in activity level, recognize accomplishments and plan to overcome challenges<br>• Describing thoughts as another modifiable contributing factor to the pain experience can be empowering
**5 –<br>Planning for and dealing with flare ups**	• Review activity log and progress towards goals during fourth week<br>• Problem solve any barriers to goals from fourth week<br>• Establish activity and participation goals for fifth week<br>• Review thought monitoring and self-talk strategies discussed last week and evaluate successes and barriers to implementation<br>• Develop a personal plan for implementing strategies to minimize the number of flare-ups<br>• Develop a personal plan for dealing with flare-ups with emphasis on thinking about "active coping strategies first"	• Briefly review the relationships between activity, thoughts, emotions and pain<br>• Discuss potential physiological explanations for flare-ups<br>• Discuss relationship between active coping and recovery from flare-ups	• Reflect on exercises from week 4<br>• Use the reflection on week 4 to modify, maintain, or progress exercises<br>• Discuss potential progression ideas for each exercise and plotting course towards long-term exercise goals<br>• Review plan for frequent aerobic activity and modify plan if needed	• Encourage self-efficacy through development of plans for reducing the number of flare-ups and dealing for flare-ups when they occur<br>• Recognizing ability to self-monitor and independently perform self-management strategies to encourage confidence with self-management
**6 –<br>Review, progression, and self-monitoring**	• Review activity log and progress towards goals during fifth week<br>• Develop plan for future activity planning and logging<br>• Review short-and long-term goals and create an updated plan to work towards these goals<br>• Discuss self-monitoring through identifying strategies that worked well throughout the program and strategies that did not work well and developing a plan to utilize helpful strategies	• Review pain physiology and the relationships between movement, activity, stress, thoughts, emotions and pain<br>• Review multifaceted approach covered over the last 6 weeks: changes in activity levels, exercises, stress and emotional regulation, thought monitoring, self-talk, planning for and dealing with flare-ups<br>• Discuss the importance of adherence, monitoring progress, and overcoming barriers	• The exercise goal for the final week is not to add any new exercises, but to ensure confidence with the existing exercise program and to ensure participant is confident with ability to progress exercises over time as able	• The goal during the final session is to let the patient take the lead in planning for the future. It is important for the patient to feel like s/he has control and feels empowered to continue to implement strategies learned and practiced during the intervention<br>• Establishing self-monitoring strategies and plans to deal with unanticipated flare-ups or barriers will aid in building the confidence in self-management and progression
