# Supplementary material for: Depicting individual responses to physical therapist led chronic pain self-management support with pain science education and exercise in primary health care: multiple case studies
Source: Arch Physiother. 2017 Apr 20;7:4. doi: 10.1186/s40945-017-0032-x (PMC5759926; doi:10.1186/s40945-017-0032-x)
Supplement: Supplementary file 2 — Rationale for interventions, strategies, and objectives included. (PDF 304 kb) [file 40945_2017_32_MOESM2_ESM.pdf]

Additional File 2: Rationale for interventions, strategies, and objectives included

Objective or Treatment strategy	Reason for inclusion
Self-management	
Progressive goal setting	Progressive goal setting is an important way to involve the client in their own care. It can improve motivation to adhere to recommendations[1] and is an important component of the chronic care model[2]. Progressive goal setting has been an important component of many self-management programs and can contribute to improvements in adherence and function [3–5].
Graded exposure	Fear of movement and re-injury is associated with increased disability and influences prognosis in people experiencing pain[6–8]. Exposure therapy is a behavioural strategy that involves exposing a person with a fear to a feared stimuli at a low intensity and in a context that is not associated with fear with the aim of reducing or extinguishing the fear. In the context of chronic pain, graded exposure is a strategy aimed at reducing fear of movement or activity by gradually exposing the person to the feared activity in a context which does not evoke a great deal of fear. This approach has been shown to reduce pain related fear and disability[9–11].
Graded activity	Gradual increases in activity are not only effective at reducing disability in people experiencing fear of movement and fear of activity. Graded activity and graded exercise approaches have demonstrated positive benefits for others who are experiencing chronic pain as well[11–15]. Gradual increases in activity are a key component of COMMENCE and attention is drawn in the education to the effects of activity on biological, psychological, and social factors associated with pain and related disability.
Activity scheduling and activity log	People with chronic pain often understand that increased activity and exercise participation are beneficial[16]. Many even have a goal to increase activity levels. Unfortunately, there is a gap between motivation to increase activity levels and participation in increased activity due to challenges implementing behaviour changes[17–19]. A strategy that is gaining support for aiding in behaviour change is targeting implementation intentions rather than motivation intentions. Several studies have demonstrated that implementation intention interventions help reduce the gap between a motivation to change activity levels and achieving the behaviour change[17–19]. Having a regular activity schedule may help the participant plan how they will implement the change in activity level. An activity log provides an opportunity for the participant to monitor success of the implementation plan.
Encourage use of social supports	People with chronic pain who have high levels of social support from others are more likely to experience improvements in pain and disability[20]. While the number of social supports available to an individual may be challenging to modify, the use of the social supports in place is something that could be encouraged in the intervention through activity scheduling. The group setting may provide an opportunity for new social supports.
Education and discussion about healthy sleep patterns and behaviours	Sleep deprivation is a common complaint in people experiencing chronic pain[21]. COMMENCE is a client focused intervention and although most of the treatment strategies included in this intervention aim at increasing activity participation, strategies are provided to address other common complaints that are important to the participants. Strategies included in this education session include education on sleep behaviours, controlling external stimuli, sleep restriction, reducing catastrophic thinking around reduced sleep, and using increases in activity during the day to improve sleep at night[22].
Relaxation strategies	Some relaxation strategies have been shown to reduce pain and improve stress responses[23]. Traditionally, relaxation approaches have been passive in nature. However, there is reason to question whether passive relaxation approaches will have any influence on increased function and participation, which is the focus of COMMENCE. Relaxation strategies, therefore, will be framed as tools to manage symptoms in order to be able to participate in gradual increases in activity.
Self-monitoring and problem solving	Adherence to treatment is integral to the success of treatment[24]. Self-monitoring is introduced from the first day of COMMENCE to encourage active involvement of the participant and ultimately a feeling of self-efficacy. This includes monitoring completion of the activity log and progress towards goals. This monitoring encourages the participant to celebrate successes and problem solve through barriers to progress. Strategies are provided towards the end of the program to monitor progress towards longer-term goals.
Pain science education	
Pain neurophysiology education	Pain neurophysiology education is included based on its demonstrated ability to reduce pain and improve function in people experiencing pain when applied alongside active rehabilitation. For example, in people with low back pain, intensive education that emphasizes cognitive-behavioural or neurophysiological aspects of pain have demonstrated improvements in pain, disability, health-care utilization, self-efficacy and negative pain cognitions[25, 26].

Education and discussion about biological, psychological, and social influences on pain	A scientific lay explanation of biological, psychological, and social factors associated with pain may help the participant understand some of their symptoms, feel legitimized, and understand the rationale for some of the treatment approaches provided. Epidemiological studies have demonstrated that biological, psychological, and social factors are associated with chronic pain and disability and can predict which patients have a poor prognosis[27, 28]. People with pain are open to discussion of psychological and social influences on pain when they are presented as contributing factors to pain rather than the cause of pain[16]. Education on the many factors that influence pain are important to helping the participant understand the biopsychosocial approach encouraged in this self-management program.
Education and discussion about the lack of a linear relationship between pain and tissue damage	Misconceptions about a close or even causal relationship between tissue damage and pain are common and can contribute to the persistence of pain and disability[29–31]. These misconceptions may lead to fear of movement and activity[29]. The fear avoidance model suggests that fear of movement and activity leads to withdrawal and avoidance of participating in usual activities, which can lead to the development and maintenance of depression and disability[32, 33]. While there are limitations to this model[34], the importance of addressing fear as part of the treatment of chronic pain is evident and providing an accurate understanding of this relationship may contribute to changes in perception of movement and activity.
Education and discussion about neuroplasticity and adaptability of other systems	Higher expectations of recovery following an injury is predictive of better rehabilitation outcomes[35–37]. Teaching people with pain about neuroplasticity and adaptability of other systems may help to increase expectations of positive change through a better understanding of the means through which these changes can occur. Education regarding neuroplasticity has been a component of many of the effective pain neurophysiology education protocols[25].
Education and discussion about the relationship between stress and pain	Research continues to demonstrate close relationships between stress and pain[38, 39]. For example, presence of post-traumatic stress disorder symptoms is a prognostic indicator of a poor recovery in people with neck pain after a whiplash injury[38–40]. Discussing the relationship between stress and pain may help the participant understand the relationship as well as ‘how and why’ some of the interventions included in this treatment program, such as stress reduction strategies, can influence pain and disability.
Education and discussion about the relationship between thoughts and pain	The concept that negative cognitions can contribute to the onset or maintenance of the pain experience or associated disability is well established[7, 41, 42]. Helping people understand these relationships is important to helping them reduce negative cognitions that may pose barriers to participation in usual activities. This discussion helps to introduce treatment strategies such as thought monitoring, self-talk and graded exposure.
Education and discussion about the relationship between emotions and pain	The International Association for the Study of Pain (IASP) defines pain as: “an unpleasant sensory and emotional experience associated with actual or potential tissue damage or described in terms of such damage[43].” Pain itself is an emotional experience, but there are close relationships between other emotions and pain. Depressive symptoms are commonly associated with chronic pain[44, 45]. Anger and frustration may also be associated with more intense pain and greater disability[46, 47]. Participants are encouraged to identify positive and negative emotions and understand how they can influence pain. Discussion focuses on how they can modify these potential contributing factors through gradual increases in activity and participation.
Use of stories and metaphors	The use of analogies and metaphors as a means of teaching complex science has been discussed for a long time[48]. Only recently has this method of communication been discussed in the area of teaching people about pain. One randomized trial suggests using stories and metaphors to teach people with pain about pain is an effective strategy to change pain related beliefs[49]. The education included in COMMENCE is complex and therefore it is important that the messages are delivered in an engaging and easy to understand way.
Exercise	
Movement and exercise that does not increase pain intensity	In healthy populations, people often experience an analgesic effect from participation in movement and activity, especially moderate-high intensity aerobic exercise[50, 51]. It is hypothesized that this analgesic effect is associated with release of endogenous opioids, release of growth factors, and changes in top down inhibition of nociceptive input[52, 53]. In people who have been experiencing pain for an extended period of time, this analgesic effect is often lost[54]. Despite this, exercises have been associated with improved outcomes in people experiencing chronic pain[12, 13, 55]. It is important that patients understand these physiological changes and how increasing activity is important even though the exercises may not provide a temporary analgesic effect as they would prior to the persistence of pain. A hypothesis of COMMENCE is that it is important that at least some of the exercises start at an intensity level that does not exacerbate symptoms, so that participants can experience exercise that does not increase pain. Exercises that cause increases in pain may cause the participant to avoid physical activity, especially in

	those who are already demonstrating avoidance behaviours. Emphasis, therefore, is placed on increases in activity at a rate and intensity that do not increase symptoms for an extended period after exercise.
Progressive functional exercises	Progressive exercises have shown consistent increases in function across multiple chronic pain conditions[12–14, 55]. However, current evidence does not provide us with suggestions of the most effective type of exercise, beyond suggesting that it is important for the exercise to be region specific [12, 13, 56]. A hypothesis of COMMENCE is that exercises more specific to the functional goals of the participant will be more effective at improving functional abilities. This hypothesis has two underlying assumptions: specificity principles make goal-specific exercises more effective at changing function and goal-oriented exercises are more meaningful to participants which makes them more likely to be adhered to.
Aerobic exercise	Aerobic exercise can result in positive physiological changes to the nervous system, motor system, endocrine system, and immune system. Evidence in people with persistent pain suggests regular aerobic exercise can improve function and mood[57]. Also, it is included as a means of developing participant confidence in the ability to perform activities in gradually progressing dosages and for the other health benefits associated with regular aerobic exercise.
Cognitive behavioural principles	
Developing a strong relationship between health care provider and patient	A strong alliance between the health care provider and the patient is an important contributor to the success of rehabilitation interventions[58–60]. Specific focus is placed on developing a strong relationship between the health care provider and the participant in COMMENCE. This is accomplished with effective communication skills, which have been shown to improve patient satisfaction and adherence to behaviour change[58]. Also, health care providers are encouraged to explore their patients' beliefs, refer to the patients' beliefs in the education, and checking the understanding of the explanations provided. All of these strategies may help to develop a strong therapeutic relationship.
Encouraging disclosure	Encouraging emotional disclosure has been shown to be effective for heterogenous populations of people with pain[61, 62]. This may be particularly important in people who are catastrophic thinkers. People who score high on measures of catastrophizing have a tendency to increase their communication of the pain experience and pain behaviours until they feel that their message is received[63]. Disclosure is considered important in allowing these participants to focus on increases in activity and progress towards their goals.
Developing self-efficacy	People with chronic pain who have a higher sense of self-efficacy tend to experience better functional outcomes[64, 65]. Self-efficacy can be important to the performance and maintenance of behaviour change in people experiencing pain[66, 67]. The main mechanism through which self-efficacy will be targeted is gradual increases in performance of goal-relevant activities. A number of additional strategies are used in an attempt to maximize self-efficacy: patient-led collaborative goal setting, using activity scheduling and activity logs in order to create a plan of action and self-monitor progress towards goals, reflection on changes in activity accomplished during the program, experience problem solving through barriers to increases in activity, and reflection on independent use of the strategies provided during the program.
Reflection on changes in activity levels throughout the program	Reflection on changes in activity and participation is encouraged throughout the program. People with misconceptions about pain such as catastrophic thinking and over-predicting pain with activity may be encouraged to change these misconceptions if they participate in increases in activity without exacerbating symptoms and recognize these successes. Also, reflection on changes in activity could influence self-efficacy and participation in life-role activities through re-evaluation of current abilities.
Thought monitoring	Cognitive behavioural approaches have been demonstrated to contribute to reducing negative cognitions such as catastrophic thinking[68]. People who experience reductions in catastrophic thinking are more likely to improve in response to treatment[69]. It has been suggested that in order to reduce catastrophic thinking, one should first help the person understand that negative thinking can have negative effects on emotions, behaviour, and function. The therapist can help the participant identify when he or she is experiencing negative thoughts that may impact behaviour and help the participant distance him/herself from those negative thoughts[70]. This is encouraged through thought monitoring.
Helpful Self-talk	Changing negative self-talk to positive self-talk and self-reassurance can be a helpful tool to provide positive reinforcement along-side increases in activity. An example of changing self-talk could include shifting from, "This hurts too much, I will never get it done" to "This is getting sore, but I know if I break up the task, I can get it done, and I will feel better having accomplished it." Another example of self-talk could include shifting from, "This is really painful, something very serious must be going on" to "This is painful, but I know it is safe to continue."

References

1. Locke E a, Latham GP: Building a practically useful theory of goal setting and task motivation. A 35-year odyssey. *Am Psychol* 2002, 57:705–717.

2. Bodenheimer T: Patient Self-management of Chronic Disease in Primary Care. *JAMA* 2002, 288:2469.

3. Stewart MJ, Maher CG, Refshauge KM, Herbert RD, Bogduk N, Nicholas M: Randomized controlled trial of exercise for chronic whiplash-associated disorders. *Pain* 2007, 128:59–68.

4. Christiansen S, Oettingen G, Dahme B, Klinger R: A short goal-pursuit intervention to improve physical capacity: A randomized clinical trial in chronic back pain patients. *Pain* 2010, 149:444–452.

5. Coppack RJ, Kristensen J, Karageorghis CI: Use of a goal setting intervention to increase adherence to low back pain rehabilitation: a randomized controlled trial. *Clin Rehabil* 2012, 26:1032–42.

6. Swinkels-Meewisse IEJ, Roelofs J, Schouten EGW, Verbeek ALM, Oostendorp RAB, Vlaeyen JWS: Fear of movement/(re)injury predicting chronic disabling low back pain: a prospective inception cohort study. *Spine (Phila Pa 1976)* 2006, 31:658–64.

7. George SZ, Stryker SE: Fear-avoidance beliefs and clinical outcomes for patients seeking outpatient physical therapy for musculoskeletal pain conditions. *J Orthop Sports Phys Ther* 2011, 41:249–259.

8. Crombez G, Vlaeyen JWS, Heuts PHTG, Lysens R: Pain-related fear is more disabeling than pain itself: evidence on the role of pain-related fear in chronic back pain disability. *Pain* 1999, 80:329–339.

9. de Jong JR, Vlaeyen JWS, van Eijsden M, Loo C, Onghena P: Reduction of pain-related fear and increased function and participation in work-related upper extremity pain (WRUEP): effects of exposure in vivo. *Pain* 2012, 153:2109–18.

10. Vlaeyen JWS, De Jong JR, Onghena P, Kerckhoffs-Hanssen M, Kole-Snijders AMJ: Can pain-related fear be reduced? The application of cognitive-behavioural exposure in vivo. *Pain Res Manag* 2002, 7:144–53.

11. George SZ, Wittmer VT, Fillingim RB, Robinson ME: Comparison of graded exercise and graded exposure clinical outcomes for patients with chronic low back pain. *J Orthop Sports Phys Ther* 2010, 40:694–704.

12. Gross A, Kay TM, Paquin J-P, Blanchette S, Lalonde P, Christie T, Dupont G, Graham N, Burnie SJ, Gelley G, Goldsmith CH, Forget M, Hoving JL, Brønfort G, Santaguida PL: Exercises for mechanical neck disorders. *Cochrane database Syst Rev* 2015, 1:CD004250.

13. Searle A, Spink M, Ho A, Chuter V: Exercise interventions for the treatment of chronic low back pain: A systematic review and meta-analysis of randomised controlled trials. *Clin Rehabil* 2015:0269215515570379–.

14. van Middelkoop M, Rubinstein SM, Verhagen AP, Ostelo RW, Koes BW, van Tulder MW: Exercise therapy for chronic nonspecific low-back pain. *Best Pract Res Clin Rheumatol* 2010, 24:193–204.

15. Leeuw M, Goossens MEJB, van Breukelen GJP, de Jong JR, Heuts PHTG, Smeets RJEM, Köke AJA, Vlaeyen JWS: Exposure in vivo versus operant graded activity in chronic low back pain patients: results of a randomized controlled trial. *Pain* 2008, 138:192–207.

16. Macdermid JC, Walton DM, Miller J: What is the Experience of Receiving Health Care for Neck Pain? *Open Orthop J* 2013, 7:428–39.

17. Luszczynska A, Sobczyk A, Abraham C: Planning to lose weight: randomized controlled trial of an implementation intention prompt to enhance weight reduction among overweight and obese women. *Health Psychol* 2007, 26:507–12.

18. Luszczynska A: An implementation intentions intervention, the use of a planning strategy, and physical activity after myocardial infarction. *Soc Sci Med* 2006, 62:900–8.

19. Latimer AE, Ginis KAM, Arbour KP: The efficacy of an implementation intention intervention for promoting physical activity among individuals with spinal cord injury: A randomized controlled trial. .

20. Hurwitz EL, Goldstein MS, Morgenstern H, Chiang L-M: The impact of psychosocial factors on neck pain and disability outcomes among primary care patients: results from the UCLA Neck Pain Study. *Disabil Rehabil* 2006, 28:1319–29.

21. Artner J, Cakir B, Spiekermann J-A, Kurz S, Leucht F, Reichel H, Lattig F: Prevalence of sleep deprivation in patients with chronic neck and back pain: a retrospective evaluation of 1016 patients. *J Pain Res* 2013, 6:1–6.

22. Finan PH, Buenaver LF, Coryell VT, Smith MT: Cognitive-Behavioral Therapy for Comorbid Insomnia and Chronic Pain. *Sleep Med Clin* 2014, 9:261–274.

23. Meeus M, Nijs J, Vanderheiden T, Baert I, Descheemaeker F, Struyf F: The effect of relaxation therapy on autonomic functioning, symptoms and daily functioning, in patients with chronic fatigue syndrome or fibromyalgia: a systematic review. *Clin Rehabil* 2015, 29:221–33.

24. Nicholas MK, Asghari A, Corbett M, Smeets RJEM, Wood BM, Overton S, Perry C, Tonkin LE, Beeston L: Is adherence to pain self-management strategies associated with improved pain, depression and disability in those with disabling chronic pain? *Eur J Pain* 2012, 16:93–104.

25. Louw A, Diener I, Butler DS, Puentedura EJ: The effect of neuroscience education on pain, disability, anxiety, and stress in chronic musculoskeletal pain. *Arch Phys Med Rehabil* 2011, 92:2041–2056.

26. Linton SJ, Andersson T: Can chronic disability be prevented? A randomized trial of a cognitive-behavior intervention and two forms of information for patients with spinal pain. *Spine (Phila Pa 1976)* 2000, 25:2825–31; discussion 2824.

27. Walton DM: Risk Factors for Persistent Problems Following Whiplash Injury: Results of a Systematic Review and Meta-Analysis. *J Orthop Sports Phys Ther* 2008, 43:31–43.

28. Sterling M, Jull G, Kenardy J: Physical and psychological factors maintain long-term predictive capacity post-whiplash injury. *Pain* 2006, 122:102–108.

29. Stenberg G, Fjellman-Wiklund A, Ahlgren C: "I am afraid to make the damage worse"--fear of engaging in physical activity among patients with neck or back pain--a gender perspective. *Scand J Caring Sci* 2014, 28:146–54.

30. Buitenhuis J, de Jong PJ, Jaspers JPC, Groothoff JW: Catastrophizing and causal beliefs in whiplash. *Spine (Phila Pa 1976)* 2008, 33:2427–2433; discussion 2434.

31. Vargas-Prada S, Martínez JM, Coggon D, Delclos G, Benavides FG, Serra C: Health beliefs, low mood, and somatizing tendency: contribution to incidence and persistence of musculoskeletal pain with and without reported disability. *Scand J Work Environ Health* 2013, 39:589–98.

32. Vlaeyen JW, Linton SJ: Fear-avoidance and its consequences in chronic musculoskeletal pain: a state of the art. *Pain* 2000, 85:317–32.

33. Crombez G, Eccleston C, Van Damme S, Vlaeyen JWS, Karoly P: Fear-avoidance model of chronic pain: the next generation. *Clin J Pain* 2012, 28:475–83.

34. Wideman TH, Adams H, Sullivan MJL: A prospective sequential analysis of the fear-avoidance model of pain. *Pain* 2009, 145:45–51.

35. Iles R a., Davidson M, Taylor NF, O'Halloran P: Systematic review of the ability of recovery expectations to predict outcomes in non-chronic non-specific low back pain. *J Occup Rehabil* 2009, 19:25–40.

36. Carroll LJ, Hogg-Johnson S, van der Velde G, Haldeman S, Holm LW, Carragee EJ, Hurwitz EL, Côté P, Nordin M, Peloso PM, Guzman J, Cassidy JD: Course and Prognostic Factors for Neck Pain in the General Population. Results of the Bone and Joint Decade 2000-2010 Task Force on Neck Pain and Its Associated Disorders. *J Manipulative Physiol Ther* 2009, 32(2 SUPPL.):S87–S96.

37. Bostick GP, Carroll LJ, Brown CA, Harley D, Gross DP: Predictive capacity of pain beliefs and catastrophizing in Whiplash Associated Disorder. *Injury* 2013, 44:1465–1471.

38. McLean SA: The potential contribution of stress systems to the transition to chronic whiplash-associated disorders. *Spine (Phila Pa 1976)* 2011, 36(25 Suppl):S226–32.

39. Sterling M, Hendrikz J, Kenardy J: Similar factors predict disability and posttraumatic stress disorder trajectories after whiplash injury. *Pain* 2011, 152:1272–1278.

40. Bortsov A V, Platts-Mills TF, Peak DA, Jones JS, Swor RA, Domeier RM, Lee DC, Rathlev NK, Hendry PL, Fillingim RB, McLean SA: Pain distribution and predictors of widespread pain in the immediate aftermath of motor vehicle collision. *Eur J Pain* 2013, 17:1243–51.

41. Sullivan MJL, Bishop S, Pivik J: The pain catastrophizing scale: development and validation. *Psychol Assess* 1995, 7:432–524.

42. Sullivan MJL, Scott W, Trost Z: Perceived injustice: a risk factor for problematic pain outcomes. *Clin J Pain* 2012, 28:484–8.

43. IASP Task Force on Taxonomy: Part III: Pain terms, a current list with definitions and notes on usage. In *Classification of Chronic Pain*. Second Edi. Edited by Merskey K, Bogduk N. Seattle, Washington: IASP Press; 1994:209–214.

44. Campbell LC, Clauw DJ, Keefe FJ: Persistent pain and depression: a biopsychosocial perspective. *Biol Psychiatry* 2003, 54:399–409.

45. Chopra K, Arora V: An intricate relationship between pain and depression: clinical correlates, coactivation factors and therapeutic targets. *Expert Opin Ther Targets* 2014, 18:159–76.

46. Burns JW, Bruehl S: Anger management style, opioid analgesic use, and chronic pain severity: a test of the opioid-deficit hypothesis. *J Behav Med* 2005, 28:555–63.

47. Bruehl S, Chung OY, Burns JW, Biridepalli S: The association between anger expression and chronic pain intensity: evidence for partial mediation by endogenous opioid dysfunction. *Pain* 2003, 106:317–24.

48. Cook SH, Frances Gordon M: Teaching qualitative research: a metaphorical approach. *J Adv Nurs* 2004, 47:649–55.

49. Gallagher L, McAuley J, Moseley GL: A randomized-controlled trial of using a book of metaphors to reconceptualize pain and decrease catastrophizing in people with chronic pain. *Clin J Pain* 2013, 29:20–5.

50. Koltyn KF: Exercise-induced hypoalgesia and intensity of exercise. *Sports Med* 2002, 32:477–87.

51. Naugle KM, Fillingim RB, Riley JL: A meta-analytic review of the hypoalgesic effects of exercise. *J Pain* 2012, 13:1139–50.

52. Koltyn KF, Brellenthin AG, Cook DB, Sehgal N, Hillard C: Mechanisms of exercise-induced hypoalgesia. *J Pain* 2014, 15:1294–1304.

53. Ray CA, Carter JR: Central modulation of exercise-induced muscle pain in humans. *J Physiol* 2007, 585(Pt 1):287–94.

54. Nijs J, Kosek E, Van Oosterwijck J, Meeus M: Dysfunctional endogenous analgesia during exercise in patients with chronic pain: to exercise or not to exercise? *Pain Physician* 2012, 15(3 Suppl):ES205–13.

55. Busch AJ, Schachter CL, Overend TJ, Peloso PM, Barber K a R: Exercise for fibromyalgia: A systematic review. *J Rheumatol* 2008, 35:1130–1144.

56. Van Middelkoop M, Rubinstein SM, Kuijpers T, Verhagen AP, Ostelo R, Koes BW, Van Tulder MW: A systematic review on the effectiveness of physical and rehabilitation interventions for chronic non-specific low back pain. *Eur Spine J* 2011, 20:19–39.

57. O'Connor SR, Tully MA, Ryan B, Bleakley CM, Baxter GD, Bradley JM, McDonough SM: Walking Exercise for Chronic Musculoskeletal Pain: Systematic Review and Meta-Analysis. *Arch Phys Med Rehabil* 2014, 96:724–734.e3.

58. Hall AM, Ferreira PH, Maher CG, Latimer J, Ferreira ML: The influence of the therapist-patient relationship on treatment outcome in physical rehabilitation: a systematic review. *Phys Ther* 2010, 90:1099–110.

59. Ferreira PH, Ferreira ML, Maher CG, Refshauge KM, Latimer J, Adams RD: The therapeutic alliance between clinicians and patients predicts outcome in chronic low back pain. *Phys Ther* 2013, 93:470–8.

60. Fuentes J, Armijo-Olivo S, Funabashi M, Miciak M, Dick B, Warren S, Rashiq S, Magee DJ, Gross DP: Enhanced therapeutic alliance modulates pain intensity and muscle pain sensitivity in patients with chronic low back pain: an experimental controlled study. *Phys Ther* 2014, 94:477–89.

61. Lumley MA, Sklar ER, Carty JN: Emotional disclosure interventions for chronic pain: from the laboratory to the clinic. *Transl Behav Med* 2012, 2:73–81.

62. Gillis ME, Lumley MA, Mosley-Williams A, Leisen JCC, Roehrs T: The health effects of at-home written emotional disclosure in fibromyalgia: a randomized trial. *Ann Behav Med* 2006, 32:135–46.

63. Sullivan MJL, Martel MO, Tripp D, Savard A, Crombez G: The relation between catastrophizing and the communication of pain experience. *Pain* 2006, 122:282–8.

64. Wright A a, Cook CE, Flynn TW, Baxter GD, Abbott JH: Predictors of response to physical therapy intervention in patients with primary hip osteoarthritis. *Phys Ther* 2011, 91:510–524.

65. Miles CL, Pincus T, Carnes D, Homer KE, Taylor SJC, Bremner S a., Rahman A, Underwood M: Can we identify how programmes aimed at promoting self-management in musculoskeletal pain work and who benefits? A systematic review of sub-group analysis within RCTs. *Eur J Pain* 2011, 15.

66. Jensen MP, Nielson WR, Kerns RD: Toward the development of a motivational model of pain self-management. *J Pain* 2003, 4:477–92.

67. Rhodes RE, Plotnikoff RC, Courneya KS: Predicting the physical activity intention-behavior profiles of adopters and maintainers using three social cognition models. *Ann Behav Med* 2008, 36:244–52.

68. Williams AC de C, Eccleston C, Morley S: Psychological therapies for the management of chronic pain (excluding headache) in adults. *Cochrane database Syst Rev* 2012, 11:CD007407.

69. Burns JW, Kubilus A, Bruehl S, Harden RN, Lofland K: Do changes in cognitive factors influence outcome following multidisciplinary treatment for chronic

pain? A cross-lagged panel analysis. *J Consult Clin Psychol* 2003, 71:81–91.

70. Turk D, Meichenbaum D, Genest M: *Pain and Behavioural Medicine: A Cognitive Behavioural Perspective*. New York: Guilford; 1983.
